# A New Urease Inhibitor from *Viola betonicifolia*

**DOI:** 10.3390/molecules191016770

**Published:** 2014-10-17

**Authors:** Naveed Muhammad, Muhammad Saeed, Ajmal Khan, Achyut Adhikari, Abdul Wadood, Khalid Mohammed Khan, Vincenzo De Feo

**Affiliations:** 1 Department of Pharmacy, Abdul Wali Khan University, Mardan 23200, Pakistan; E-Mail: drnaveedrph@gmail.com; 2 Department of Pharmacy, University of Peshawar, Peshawar 25120, Pakistan; E-Mail: saeedrph2000@yahoo.com; 3 H. E. J. Research Institute of Chemistry, International Center for Chemical and Biological Sciences, University of Karachi, Karachi 75270, Pakistan; E-Mails: ajmalchemist@yahoo.com (A.K.); palpaliachyut@yahoo.com (A.A.); khalid.khan@iccs.edu (K.M.K.); 4 Computational Medicinal Chemistry Laboratory, Department of Biochemistry, Shankar Campus, Abdul Wali Khan University, Mardan 23200, Pakistan; E-Mail: awadood@awkum.edu.pk; 5 Department of Pharmacy, University of Salerno, 84084 Fisciano, Salerno, Italy

**Keywords:** *Viola betonicifolia*, urease inhibition, kinetic study, molecular modeling

## Abstract

Urease has attracted much attention, as it is directly involved in the formation of infection stones and contributes to the pathogenesis of urolithiasis, pyelonephritis, ammonia and hepatic encephalopathy, hepatic coma and urinary catheter encrustation. Moreover, urease is the major cause of pathologies induced by *H. pylori*, such as gastritis and peptic ulcer*.* In the present work, the new natural compound, 3-methoxydalbergione, was isolated from *Viola betonicifolia.* A mechanistic study of this compound as a natural urease inhibitor was performed by using enzyme kinetics and docking studies. 3-Methoxydalbergione could be considered as a lead molecule for drugs useful in the urease associated diseases.

## 1. Introduction

*Viola betonicifolia* Sm. (Violaceace) is a perennial herb of about 8–20 cm in height, with triangular leaves and a petiole longer than the lamina. It has slender and unbranched roots with a short rhizome. The plant is diffused across different countries, like Nepal, Malaysia, Pakistan, China, Australia, India and Sri Lanka [1]. In Pakistan, *Viola betonicifolia* is traditionally used for the treatment of fever, cancer and epilepsy [2]. The plant is also suggested in the case of skin and blood diseases, sinusitis and to treat pneumonia and bronchitis [2]. The plant is also claimed to be useful in lung troubles, in cold and cough [3]. Recently, our group has reported the antioxidant, cytotoxic, phytotoxic and neuropharmacological properties of different extracts of this plant [4,5,6,7].

Urease (EC 3.3.1.5) hydrolyzes urea into ammonia and carbamate. Carbamate at physiological pH hydrolyzes spontaneously to carbonic acid and yields another molecule of ammonia [8]. Urease plays a crucial role in the pathogenesis of gastric and peptic ulcers and cancer, by facilitating the survival of *Helicobacter pylori* in the acidic environment of the stomach. It is reported that ureases also cause urolithiasis and infections by *Yersinia enterocolitica* and *Proteus mirabilis*. The ureases are also responsible for the development of infection-induced reactive arthritis and acute pyelonephritis [9,10].

The main nitrogenous waste product of biological system urea is quickly metabolized by the action of urease present in microorganisms. The urease enzymes are present in a number of fungi, bacteria and plants. Ureases play a vital role in the nitrogen cycle, as they supply nitrogen for the growth of microorganisms by catalyzing urea degradation [11]. In agriculture, a high level of urease activity is associated with several environmental and economic hazards. Globally, urea is commonly used as a fertilizer. In agriculture, the high activity of ureolytic bacteria increases the amount of ammonia in the soil via fast urea degradation. Firstly, the plants are damaged due to a lack of necessary nutrients, and secondly, the plants are damaged by the toxicity of ammonia and carbon dioxide, which are released from urea degradation [11]. During the seed germination process, urease plays a vital role in the metabolism of nitrogen, helping many soil microorganisms to obtain nitrogen for their growth [12,13].

The discovery of effective and safe urease inhibitors is an important area of pharmaceutical research due to the association of ureases with several pathological conditions, as well as for agriculture applications [14]. A number of synthetic urease inhibitors, such as hydroxyurea, formamide, β-mercaptoethanol, acetohydroxamic acid, phosphoric triamide, boric acid, bismuth derivatives, quinones, triazoles, thiazoles, ketoacids, coumarins and thiobarbituric acids, are known, but few studies have been carried out on natural inhibitors [13,15,16,17,18].

In the present study, we have isolated the new compound, 3-methoxydalbergione, from *Viola betonicifolia* and studied its possible urease inhibitory activity. Kinetic and molecular modeling studies were performed to understand the mode of action of this compound.

## 2. Results and Discussion

**Figure 1:**
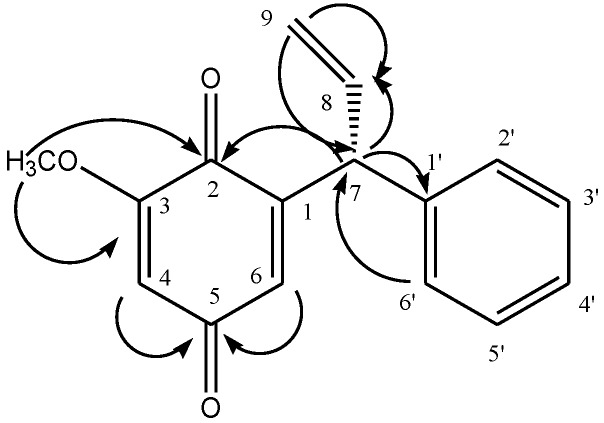
Key HMBC interactions in 3-methoxydalbergione.

Compound **1** was isolated as a yellow spongy mass from combined chloroform and ethyl acetate fractions of a methanolic extract of *Viola betonicifolia*. Its optical rotation ${\left[\alpha \right]}_{D}^{28}$ = +67 indicated the presence of a chiral center in the molecule. The UV spectrum displayed absorption maximum (λ_max_) at 206 nm and 260 nm, which implied that the compound possess a neoflavonoid structure. IR spectrum showed absorptions at 1671 cm^−1^ (conjugated ketone) and 1652 (aromatic). EIMS showed a molecular ion peak at *m/z* 254 and fragment peaks at 239, 172 and 117. The molecular formula of Compound **1**, C_16_H_14_O_3_, was determined by EIMS and NMR methods. The ^1^H-NMR spectrum (Table 1) exhibited resonances at δ 4.87 (d, *J* = 8.0 Hz, H-7), 4.95 (dd, *J* = 15.0, 2.0 Hz, Ha-9), 5.22 (dd, *J* = 10.0, 2.0 Hz, Hb-9) and 6.16 (m, H-8). Other downfield signals were registered at δ 6.0 (s, H-4), 6.46 (s, H-6) and 7.19–7.22 (overlapped, H-2′-H-6′). The ^13^C-NMR spectrum (Broad Band and DEPT) (Table 1) showed resonance for all sixteen carbons, including one methyl, one methylene, nine methine and five quaternary carbons. The structure of the compound was further confirmed by using 2D-NMR techniques (HSQC, HMBC, COSY). Key HMBC interactions in Compound **1** are shown in Figure 1. Compound **1** was identified as 3-methoxydalbergione. The quinone, dalbergione, has been firstly isolated from *Dalbergia baronii* Baker (Fabaceae) [19], whereas 4-methoxydalbergione is the main allergen in *Dalbergia nigra* All. (Brazilian rosewood, palisander) and *Dalbergia latifolia* Roxb. [20] and has been isolated also from *D. retusa* Hamsl. [21].

**Table 1:** ^1^H- and ^13^C-NMR chemical shift values of Compound **1**.

C No.	δ_C_	δ_H_ (*J*, Hz)	C No	δ_C_	δ_H_ (*J*, Hz)
**1**	152.1	-	1′	141.20	-
**2**	187.8	-	2′	129.60	7.19 m
**3**	160.2	-	3′	129.70	7.22 m
**4**	108.7	6.0 s	4′	128.00	7.20 m
**5**	183.5	-	5′	129.70	7.22 m
**6**	132.6	6.46 s	6′	129.60	7.19 m
**7**	48.7	4.87 d (8.0)	OMe	56.90	3.78 m
**8**	139.2	6.16 m			
**9**	118.2	4.95 dd (15.0, 2.0) 5.22 dd (10.0, 2.0)			

**Figure 2:**
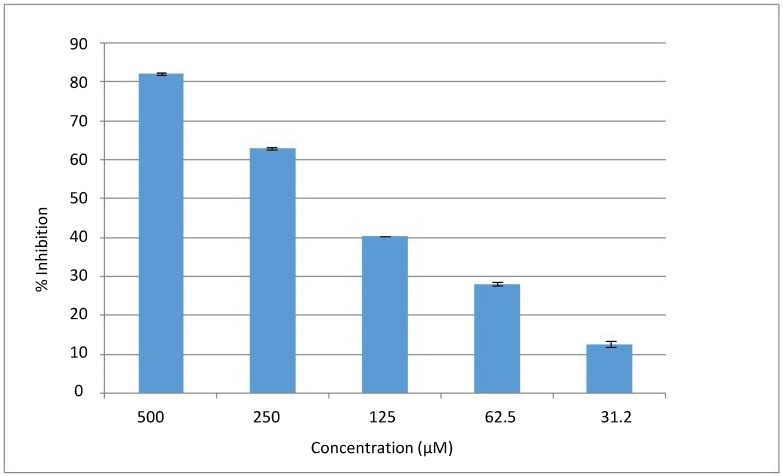
The dose response curve of Compound **1**.

Natural compounds provide a good pharmacophore template for new drugs. The compound 3-methoxydalbergione (**1**) was evaluated for its possible *in vitro* urease inhibition. The isolated compound showed a significant inhibition against urease enzyme, showing 82% inhibition at 0.5 mM, with an IC_50_ value of 169 ± 2 µM. The dose response curve is presented in Figure 2. The activity of Compound **1** might be due to the presence of a ketonic moiety that may chelate to Ni in the binding pocket of urease enzyme. Kinetics studies of this compound were performed to check its inhibition mechanism. It was clear that Compound **1** is a pure competitive type of inhibitor with a Ki value of 138.16 ± 0.06 µM.

**Figure 3:**
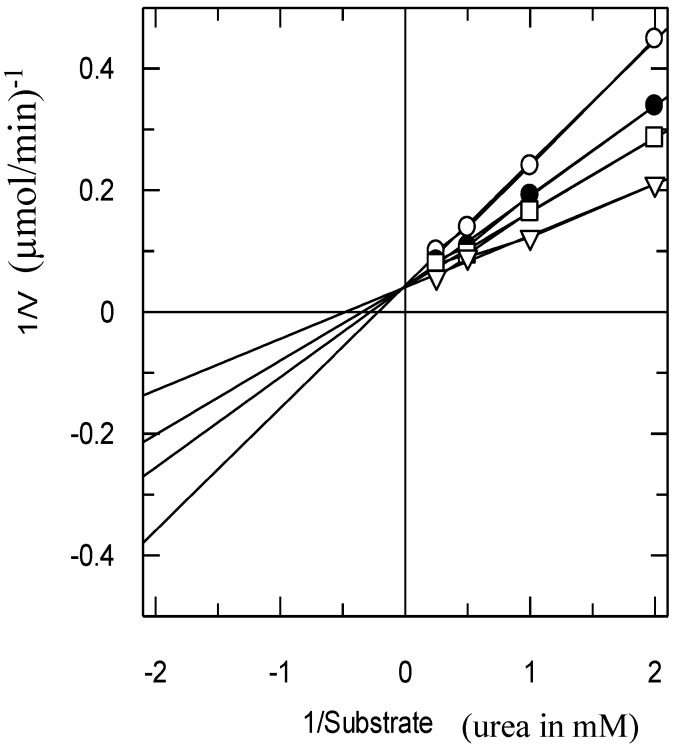
The inhibition of urease by Compound **1** using the Lineweaver–Burk plot of the reciprocal of rate of reaction (velocities in µmol/min^−1^ *versus* the reciprocal of the substrate (urea) in the absence (∆) and in the presence of 80 µM (□), 100 µM (●) and 200 µM (○) of Compound **1**.

The type of inhibition was determined by Lineweaver–Burk plots (Figure 3). The reciprocal of the rate of the reaction was plotted against the reciprocal of the substrate concentration to monitor the effect of the inhibitor on both K_m_ and V_max_. Figure 3 shows that the V_max_ of jack bean urease was not affected in the presence of different concentrations of Compound **1**, while the K_m_ increased, which indicated a pure competitive-type of inhibition.

**Figure 4:**
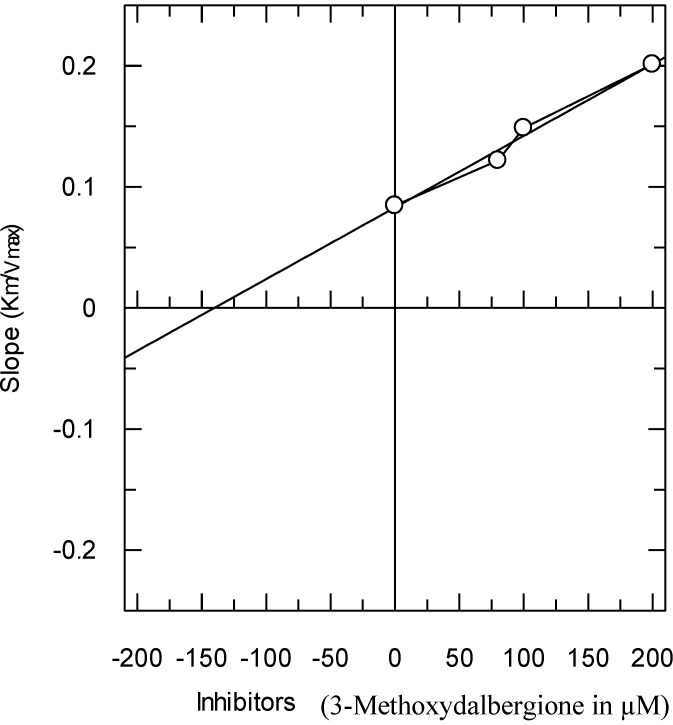
The secondary replot of the Lineweaver–Burk plot between the slopes of each line on the Lineweaver–Burk plot *versus* different concentrations (0, 80, 100 and 200 µM) of Compound **1**.

The secondary replots of the Lineweaver–Burk plots were plotted to determine the Ki values, calculated by plotting the slope of each line in the Lineweaver–Burk plots against various concentrations of Compound **1** (Figure 4). The Ki value was confirmed by a Dixon plot, by plotting the reciprocal of the rate of reaction against the different concentrations of Compound **1**.

**Figure 5:**
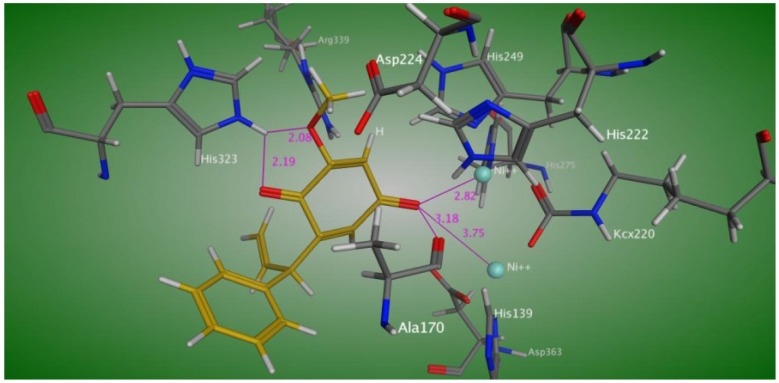
Predicted binding mode of Compound **1** in the active site of *Bacillus pasteurii* urease.

In order to understand the binding mode, Compound **1** was docked into the active site of *Bacillus pasteurii* urease. The docking study (Figure 5) showed interactions of one of the keto oxygens of the hydroquinone moiety of Compound **1** with both nickel atoms at a distance of 2.82 and 3.75 Å, respectively, and also established a hydrogen bond with Ala 170. The second keto oxygen and methoxy oxygen atoms of the hydroquinone moiety make a hydrogen bond with its His 323 at a distance of 2.08 and 2.19 Å, respectively.

## 3. Experimental Section

### 3.1. Plant Material

In April 2010, a whole plant of *V. betonicifolia* was collected from the district of Swat, Khyber Pakhtunkhawa, Pakistan. The plant was identified in the section of taxonomy, Botany Department, Peshawar University, and a specimen was in this herbarium, with the voucher number 6410/Bot.

### 3.2. Plant Extraction and Fractionation

The vegetal material (12 kg) was dried in air and powdered. The powder was extracted by maceration with methanol for 14 days with occasional shaking at room temperature. The extract was then filtered and concentrated at low temperature (45 °C), resulting in a crude methanolic extract (1.98 kg, 22% *w*/*w*). This extract (1.60 kg) was dissolved in distilled water and further fractionated into various solvents (*n-*hexane, ethyl acetate, chloroform, butanol and water), yielding *n*-hexane (706 g, 44.13% *w*/*w*), chloroform (9 g, 0.56% *w*/*w*), ethyl acetate (16 g, 1.00% *w*/*w*), butanol (265 g,16.56% w/w) and aqueous (498 g, 31.13% *w*/*w*) fractions. The chloroform and ethyl acetate fractions were combined and subjected to silica gel column chromatography. The column was eluted with an EtOAc:*n*-hexane solvent system of increasing polarity. One hundred twenty-four fraction were collected and combined for their chromatographic similarity in 12 main fractions. Compound **1** (100 mg) was obtained from main Fraction 4, eluted with 25% EtOAc/*n*-hexane.

### 3.3. Identification of Compound **1**

The structural elucidation of the isolated compound was performed by spectroscopic methods (^I^H-NMR, ^13^C-NMR, HMBC, HMQC, NOESY, COSY, HREI-MS and IR). Spectra were obtained on a Vector 22 (Bruker) Fourier transform infrared (FTIR) spectrometer, employing KBr windows with CH_2_Cl_2_ as the solvent against an air background. ^1^H-NMR and ^13^C-NMR spectra were registered on a Bruker Avance spectrometer at 400 MHz. The 2D-NMR spectra were obtained on a Bruker Avance NMR spectrometer. Mass spectral information (EI and HR-EI-MS) was calculated in electron impact mode on Finnigan MAT-312 and MAT-95 XP spectrometers, and ions are given in *m*/*z* (%).

### 3.4. Urease Inhibition Assay

A 25-µL solution of urease enzyme (1 unit/well) was mixed with 5 µL of the test compound (31.25–500 µM concentration) in phosphate buffers (concentration: 4 mM; pH 6.8) and incubated for 15 min at 30 °C in each well of 96-well plates. The urease activity was measured by adding 55 μL of urea (100 mM). The production of ammonia was computed as urease activity via the indophenol methodology, as described by Weatherburn [22]. Final volumes were maintained at 200 µL by the addition of 45 µL phenol reagent (1% *w*/*v* phenol and 0.005% *w*/*v* sodium nitroprusside) and 70 µL of alkali reagent (0.5% *w*/*v* NaOH and 0.1% active chloride NaOCl) to each well. The increase in absorbance was recorded by a microplate reader (Molecular Devices, Sunnyvale, CA, USA), at 630 nm after 50 min. The results (per min absorbance change) were read by Soft Max Pro software (Molecular Devices, Sunnyvale, CA, USA). Thiourea was used as a standard inhibitor, and the percent of inhibitions were computed as follows:

% inhibition = 100 − (OD_testwell_/OD_control_) × 100 (1)

For the IC_50_ determination, the experiment was repeated for a range of inhibitor concentrations of serial dilution of 500–31.25 µM (Figure 2).

For the kinetics assessment, the same experiment was repeated, but the urea concentrations were reciprocally changed from 0.5 to 4 mM (0.5, 1, 2 and 4 mM). The experiment was repeated for a series of urea concentrations (0.5–4 mM) in the presence of a given inhibitor concentration (80, 100, 200 µM).

### 3.5. Statistical Analysis

The EZ-Fit Enzyme Kinetics program (Perrella Scientific Inc., Amherst, MA, USA) was employed to calculate the IC_50_ values. The graphs were plotted by using the GraFit program (2010). Values of the correlation coefficients, intercepts, slopes and their standard errors were calculated by the linear regression analysis by using the same program. Each point in the constructed graphs represents the mean of the three experiments [23].

### 3.6. Molecular Docking

The molecular docking was performed by using MOE-Dock implemented in the Molecular Operating Environment (MOE), using the default parameters [24]. The X-ray crystallographic structure of *Bacillus pasteurii* (PDB Code: 4UBP) was downloaded from the protein databank [25]. All molecules of water were removed from the structure of the protein. Then, hydrogens were added, and the energy minimization was carried out using a default force field. The compound structure was modeled using the Builder program implemented in MOE. The compound structure was also energy minimized, and partial charges were calculated before docking. The molecular interactions were visualized using LIGPLOT implemented in MOE.

## 4. Conclusions

From *Viola betonicifolia*, the new natural compound, 3-methoxydalbergione, was isolated. The compound showed urease inhibitor activity, being a competitive inhibitor. 3-Methoxydalbergione can be considered as a template drug for urease associated diseases. As limited studies have been conducted on natural products for urease inhibition, the present work can result in a valuable addition to the field of drug discovery.
